# Next-Generation Advances in Prostate Cancer Imaging and Artificial Intelligence Applications

**DOI:** 10.3390/jimaging11110390

**Published:** 2025-11-03

**Authors:** Kathleen H. Miao, Julia H. Miao, Mark Finkelstein, Aritrick Chatterjee, Aytekin Oto

**Affiliations:** 1 Department of Diagnostic, Molecular, and Interventional Radiology, Icahn School of Medicine at Mount Sinai, New York, NY 10029, USA; 2 Department of Radiology, University of Chicago Medicine, Chicago, IL 60637, USA

**Keywords:** prostate cancer, artificial intelligence, multiparametric MRI, PSMA PET/CT, transrectal ultrasound, machine learning, PI-RADS

## Abstract

Prostate cancer is one of the leading causes of cancer-related morbidity and mortality worldwide, and imaging plays a critical role in its detection, localization, staging, treatment, and management. The advent of artificial intelligence (AI) has introduced transformative possibilities in prostate imaging, offering enhanced accuracy, efficiency, and consistency. This review explores the integration of AI in prostate cancer diagnostics across key imaging modalities, including multiparametric MRI (mpMRI), PSMA PET/CT, and transrectal ultrasound (TRUS). Advanced AI technologies, such as machine learning, deep learning, and radiomics, are being applied for lesion detection, risk stratification, segmentation, biopsy targeting, and treatment planning. AI-augmented systems have demonstrated the ability to support PI-RADS scoring, automate prostate and tumor segmentation, guide targeted biopsies, and optimize radiation therapy. Despite promising performance, challenges persist regarding data heterogeneity, algorithm generalizability, ethical considerations, and clinical implementation. Looking ahead, multimodal AI models integrating imaging, genomics, and clinical data hold promise for advancing precision medicine in prostate cancer care and assisting clinicians, particularly in underserved regions with limited access to specialists. Continued multidisciplinary collaboration will be essential to translate these innovations into evidence-based practice. This article explores current AI applications and future directions that are transforming prostate imaging and patient care.

## 1. Introduction

Prostate cancer is one of the leading causes of cancer-related morbidity and mortality in men worldwide [1]. Accurate imaging plays a central role in the diagnosis, risk stratification, biopsy targeting, and treatment planning for prostate cancer. Multiparametric magnetic resonance imaging (mpMRI), in particular, has emerged as a cornerstone in prostate imaging due to its ability to localize and characterize suspicious lesions [2]. However, interpretation of mpMRI remains complex, time-consuming, and subject to interobserver variability, even with the use of standardized systems such as the Prostate Imaging Reporting and Data System (PI-RADS) [3].

In recent years, artificial intelligence (AI)—including machine learning (ML), deep learning (DL), and radiomics—has shown significant promise in enhancing the performance and consistency of prostate imaging. AI algorithms have demonstrated capabilities in automating lesion detection, improving segmentation accuracy, predicting Gleason scores, and guiding targeted biopsies [4,5,6]. For instance, in the PROSTATEx Challenge and international Prostate Cancer AI (PI-CAI) challenge, AI-based systems achieved high detection accuracy, with an area under the receiver operating characteristic curve (AUC) of up to 0.91 for clinically significant prostate cancer [7], comparable to human professional performance. Various AI models including 2-D and 3-D convolutional neural networks (CNNS), deep learning, U-Net, V-Net, P-Net, random forest, and adaptive Boosting models, have been applied in prostate cancer imaging. These have aided in prostate cancer detection, classification, segmentation, volume delineation, prognosis, and biopsy guidance.

AI is increasingly being applied to various imaging modalities, such as multiparametric MRI (mpMRI), prostate-specific membrane antigen positron emission tomography/computed tomography (PSMA PET/CT), and ultrasound. Deep learning models have shown high performance in lesion segmentation and metastasis detection on PSMA PET/CT imaging detecting 93% for lesions with maximum standardized uptake values greater than 5.0 [8,9]. A number of FDA-approved AI applications in prostate cancer imaging, detection, and classification implemented in clinical practice include ProstatID (Version 2.0), Quantib Prostate (Version 3.0), and PROView (Version 1.0) using MRI images and aPROMISE (Version 1.0) using PSMA PET/CT images [10].

Despite these advances, significant barriers remain to the widespread clinical adoption of AI in prostate imaging. Challenges include the need for large, diverse, and annotated datasets; generalizability across imaging platforms; algorithmic bias; and regulatory concerns related to clinical validation and integration into existing workflows [11,12].

This review aims to provide a comprehensive overview of current and emerging applications of AI in prostate imaging. It highlights key innovations in mpMRI, PSMA PET/CT, and ultrasound; examines the methodological and clinical limitations; and discusses the future potential of AI to support precision medicine in prostate cancer care.

## 2. Overview of AI Technologies in Prostate Cancer Imaging

Artificial intelligence (AI) encompasses a spectrum of computational techniques designed to emulate human cognitive functions such as pattern recognition, learning, and decision-making. In medical imaging, AI primarily leverages machine learning (ML) and its subset, deep learning (DL), to interpret complex visual and quantitative data. These methods are particularly well-suited for prostate imaging due to the modality’s multidimensional nature and the nuanced distinctions required for clinically significant prostate cancer (csPCa) detection.

### 2.1. Machine Learning and Radiomics

Machine learning and radiomics have become central to advancing prostate cancer imaging by enabling quantitative, reproducible analysis across multiparametric MRI (mpMRI), PSMA PET, and ultrasound. In prostate cancer imaging, machine learning (ML) refers to computational methods that can identify and learn patterns from prostate imaging data. These approaches have been applied to extract and analyze radiomic features—quantitative descriptors such as texture, shape, and intensity—from prostate MRI and PET/CT scans to build predictive models, such as distinguishing between benign and malignant prostate lesions.

Radiomics has emerged as a critical bridge between medical imaging and computational analysis, providing a framework for extracting high-dimensional quantitative features from standard imaging modalities such as mpMRI, PSMA PET, and ultrasound. By transforming images into large-scale numerical datasets, radiomics can be used as input to develop machine learning models that predict tumor aggressiveness, guide biopsy decisions, or stratify risk [13,14]. For example, Algohary et al. integrated biparametric MRI radiomics including T2W and ADC maps with clinical parameters to improve diagnostic accuracy for csPCa by up to 30–80% in testing groups when compared to clinical PI-RADS performance alone [5] (Table 1). These architectures also demonstrated utility in lesion classification and Gleason score prediction.

Emerging approaches are focused on developing multi-modal AI models that integrate imaging data and radiomics with clinical, genomic, and laboratory variables [15,16,17,18]. Such integrative frameworks aim to create robust decision-support tools that account for the multifactorial nature of prostate cancer diagnosis and management [19]. Hybrid models combining radiomic and deep learning features have shown superior performance in predicting extracapsular extension, Gleason upgrading, and biochemical recurrence after treatment [20]. For example, Gong et al. demonstrated that radiomics models based on MRI showed statistically significant results (*p* < 0.001) to detect high grade PCa preoperatively with AUCs up to 0.801 using DWI models [21]. These efforts align with the broader shift toward precision medicine, wherein AI can facilitate tailored treatment strategies based on individual patient profiles.

In particular, deep learning architectures—a type of machine learning—applied to radiomics are especially relevant in prostate imaging, as they allow automated feature learning directly from imaging data rather than relying solely on handcrafted descriptors. Radiomics pipelines benefit from these architectures because deep networks can either augment handcrafted feature sets or replace them by automatically discovering high-dimensional representations that are more discriminative. Deep learning applied to radiomics enables end-to-end learning that accounts for the complex spatial heterogeneity of prostate tumors, improving reproducibility and reducing reliance on subjective manual feature engineering. This integration has shifted the field from conventional radiomic pipelines toward end-to-end frameworks that can capture complex spatial and textural relationships across mpMRI and PET, thereby enhancing diagnostic accuracy and reproducibility. For example, a deep radiomics model developed by Nketiah et al. trained on multicenter MRI patient data achieved an area under the ROC of 0.91 for diagnosing clinically significant prostate cancer—matching clinical PI-RADS performance [22] (Table 1).

Despite the advantages of radiomics and AI in prostate cancer imaging, including enhanced lesion detection, objective feature quantification, and improved prognostic modeling, several disadvantages remain. Limitations include lack of standardization, high sensitivity to variations in image quality and segmentation accuracy, and feature overfitting especially with small datasets. Nevertheless, radiomics is a rapidly emerging field and its implementation into the clinical workflow holds promise.

**Table 1:** Summary of Prostate Cancer Imaging and Overview of AI Application Studies.

Study	Imaging Modality	AI Technologies	Results
Gong et al., 2020 [21]	MRI	Deep radiomics model	Detecting high grade PCa preoperatively with AUCs up to 0.801 using DWI models
Nketiah et al., 2024 [22]	MRI	Deep radiomics model	Diagnosing clinically significant prostate cancer with an area under the ROC of 0.91
Schelb et al., 2021 [23]	MRI	U-Net model	Improving positive predictive value up to 68% for the coincidence of PI-RADs greater than or equal to 4
Lindgren Belal et al., 2023 [24]	PSMA PET/CT	CNN model	Detecting prostatic lesions in patients with newly diagnosed prostate cancer or suspected recurrence after treatment with average sensitivity of 79%
Zhao et al., 2020 [25]	MRI	U-Net model	Detecting metastatic bone lesions and pelvic lymph node lesions with sensitivity up to 99% and 90% respectively
Sun et al., 2023 [26]	TRUS	2-D and 3-D CNN P-Net models	Detecting lesions on TRUS video with diagnostic performance of 0.85–0.89

### 2.2. Deep Learning and Neural Networks

Deep learning and neural network applications in prostate cancer imaging are rapidly evolving, driving significant advances in disease detection, characterization, and treatment planning in the clinical setting [23,24]. Deep learning utilizes artificial neural networks (ANNs), especially convolutional neural networks (CNNs), to learn hierarchical patterns from imaging data. These architectures are capable of automatic feature extraction and feature learning, surpassing conventional ML in image classification, segmentation, and detection tasks [15]. Deep learning architectures are well suited to prostate cancer imaging because of their ability to capture subtle, multi-scale features within heterogeneous tissue environments. CNNs, for example, excel at extracting spatial hierarchies of features from mpMRI, such as intensity, shape, and texture, which are essential for differentiating between benign and malignant lesions. Architectures with residual or dense connections mitigate vanishing gradient problems and allow deeper models to learn more complex relationships, making them particularly effective for segmenting the prostate gland and localizing lesions [16]. For example, a CNN-based model was developed by Lindgren Belal et al., detecting prostatic lesions in 660 PSMA PET/CT scans in patients with newly diagnosed prostate cancer or suspected to have recurrence after treatment with an average sensitivity of 79% [24] (Table 1), comparable with expert review.

Traditional 2D CNNs process imaging data slice-by-slice, analyzing pixel-level patterns in two dimensions. While effective for learning spatial features within each image plane, 2D CNNs are limited in their ability to fully capture volumetric information, which is crucial for understanding the three-dimensional morphology of prostate tumors. In contrast, 3D CNNs extend convolutional kernels into three dimensions, thereby directly incorporating inter-slice contextual information and improving performance in volumetric tasks such as gland segmentation, tumor delineation, and treatment planning [24]. For example, a tool implemented in clinical practice includes the FDA-approved ProstatID, which incorporates 3-D CNN models trained on deep learning and MRI imaging data to automatically segment the prostate and detects suspicious lesions [10]. Another FDA-approved tool, Quantib Prostate, is also implemented in the clinical setting combining deep learning with MRI scans to detect prostate lesions [10].

Among the most widely adopted architectures in prostate imaging are U-Net and V-Net, both designed for biomedical segmentation tasks. U-Net is based on an encoder–decoder architecture that applies 2D convolutions with symmetric skip connections. The encoder progressively down-samples the image to extract hierarchical features, while the decoder up-samples to reconstruct the segmentation map. The skip connections bridge corresponding encoder and decoder layers, ensuring the preservation of fine spatial details—a critical factor for delineating small or irregularly shaped prostate lesions [24]. By contrast, V-Net extends this concept into the volumetric domain. It is a 3D CNN that applies three-dimensional convolutions, pooling, and deconvolutions throughout the network. V-Net employs residual connections within its encoding pathway and Dice loss as the optimization function, making it particularly effective for highly imbalanced datasets often seen in prostate cancer segmentation tasks.

In prostate MRI, for example, 2D CNNs trained on T2-weighted, diffusion-weighted imaging (DWI), and dynamic contrast-enhanced (DCE) sequences have shown strong performance in lesion detection and PI-RADS scoring. More advanced 3D CNNs, such as V-Net architectures, extend analysis into volumetric space, allowing for automated prostate gland and lesion segmentation with accuracy approaching expert performance [27,28,29].

CNN-based architectures remain the most established for segmentation and lesion detection, while hybrid radiomics–deep learning pipelines and transformer-based networks are emerging as state-of-the-art for prostate cancer classification and multimodal integration [30]. The architectural choice often depends on the clinical task: U-Net variants dominate segmentation, multi-stream CNNs perform well in multiparametric fusion, and transformer-based architectures appear most promising for generalizable multimodal analysis.

### 2.3. Multi-Modal Integration Models

Multimodal integration of multiple imaging modalities has improved prostate cancer detection along with applications in AI. Multi-modal integration models such as PSMA PET with CT has significantly improved prostate lesions detection. For example, a FDA-approved tool implemented in clinical practice, aPROMISE, can identify and quantitatively analyze suspicious prostate lesions on PSMA PET/CT using deep learning and CNN-based architecture [10]. In addition to the combination of PSMA PET with CT in diagnosis prostate lesions, the combination of multiparametric MRI (mpMRI) and PSMA PET has demonstrated substantial benefits in prostate cancer detection, localization, and staging. mpMRI provides detailed anatomic and functional information, particularly for local tumor assessment, while PSMA PET offers molecular sensitivity to detect primary and metastatic lesions with high specificity. Combining these modalities improves diagnostic accuracy and reduces false negatives compared to either technique alone, resulting in high sensitivity and high specific for clinically significant prostate cancer detection [31].

A pivotal trial, PRIMARY study, demonstrated that the addition of PSMA PET to MRI resulted in improved sensitivity (97% for combined vs. 83% for MRI alone) and negative predictive value (91% for combined vs. 72% for MRI alone) for clinically significant prostate cancer in a MRI triaged patient population [32]. Similarly, another study found that the combination of PSMA PET with MRI performed better than MRI alone, improving sensitivity (89% for combined vs. 76% for MRI alone) without affecting the specificity, especially for diagnosing clinically significant prostate cancer in PI-RADS 3 lesions [33]. These findings underscore the clinical utility of multimodal imaging in improving patient selection for biopsy, guiding focal therapy, and enhancing radiation therapy planning.

AI-driven approaches—particularly deep learning and radiomics—facilitate automated feature extraction, cross-modality correlation, and predictive modeling. For example, radiomics models combining mpMRI and PSMA PET features can improve risk stratification and Gleason grade prediction beyond what either modality can achieve alone. AI-based multimodal fusion frameworks also support automated lesion detection, reduce inter-reader variability, and can integrate imaging with clinical and genomic data to build precision oncology decision-support systems. In addition, recurrent architectures and hybrid CNN-transformer networks can capture temporal and contextual dependencies across imaging sequences, supporting lesion classification and risk prediction.

## 3. MRI, PSMA PET/CT, and Ultrasound in Prostate Cancer Imaging and Specific AI Applications

Artificial intelligence has become increasingly embedded in the clinical workflow of prostate imaging, particularly in the interpretation and analysis of MRI, PSMA PET/CT, and ultrasound. These modalities are essential for accurate prostate cancer detection, staging, and treatment planning, and AI-driven tools have shown considerable potential in improving diagnostic performance, automating complex tasks, and supporting real-time clinical decisions.

**Figure 1:**
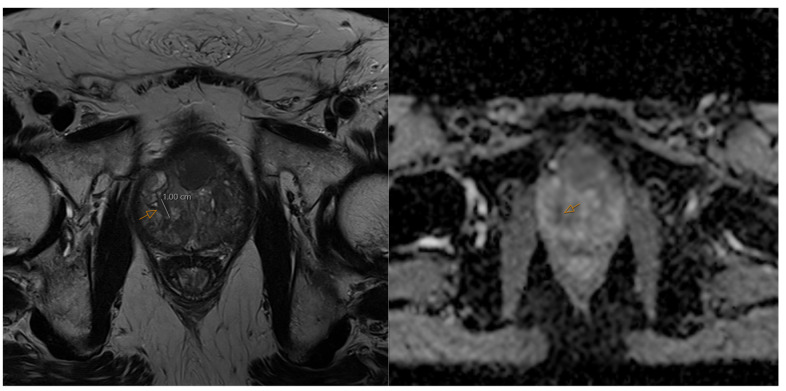
In this mpMRI prostate, the arrow shows on axial T2 sequence (**left**) and an axial DWI sequence (**right**) a 1.0 × 0.8 cm right posterior apex transitional zone Gleason 6 lesion in a 69-year-old prostate cancer patient with diabetes.

In the realm of mpMRI, which remains the gold standard for prostate cancer localization, artificial intelligence has demonstrated significant utility in lesion detection, segmentation, and classification. Figure 1 demonstrates a prostate cancer lesion on mpMRI imaging. Deep learning models, particularly CNNs, trained on large-scale, annotated mpMRI datasets, have achieved high diagnostic performance. One multicenter study by Schelb et al. reported a U-Net CNN achieving similar clinical performance to clinical PI-RADS evaluation demonstrating that in the testing set, the coincidence of PI-RADs greater than or equal to 4 with U-Net lesions improved positive predictive value up to 68% [23] (Table 1). The U-Net approach was selected because of its advantage in skip connections bridging corresponding encoder and decoder layers, preserving spatial information critical in highlighting small or irregularly shaped prostate lesions. Beyond lesion detection, AI has also improved anatomical and pathological segmentation. Automated segmentation algorithms have successfully delineated prostate zones and tumor regions with high accuracy, achieving Dice similarity coefficients exceeding 0.85, which significantly reduces both manual workload and clinical inter-reader variability [16].

AI applications in mpMRI extend to risk stratification and prediction of pathological outcomes. Radiomics-based models that extract quantitative imaging features from T2-weighted and diffusion-weighted sequences have demonstrated the ability to predict Gleason scores and stratify patients on active surveillance [5]. Such tools may eventually reduce the need for invasive biopsies by enabling more precise identification of clinically significant disease. FDA-approved AI tools in clinical practice such as Quantib Prostate (Version 3.0), ProstatID (Version 2.0), and QUIBIM Precision Prostate (Version 2.0), use MRI prostate imaging and incorporate 3-D CNN and deep learning models to automatically segment the prostate and localize suspicious lesions [10]. Furthermore, AI-assisted tools are being used in biopsy and treatment planning by optimizing target selection and mapping tumor burden, thereby enhancing diagnostic yield and minimizing unnecessary sampling [24].

**Figure 2:**
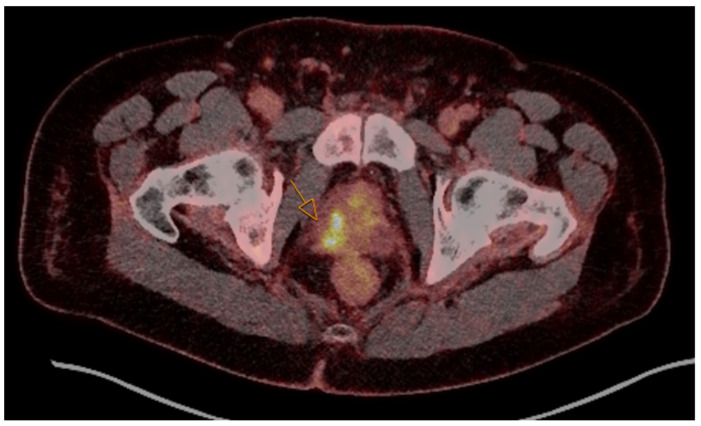
In this axial-fused PSMA PET/CT, the arrow shows a scapular focus of increased radiotracer uptake involving the right posterior peripheral zone of the base and mid gland with extension of malignancy in the base of the right seminal vesicle in a 74-year-old prostate cancer patient with hypertension and diabetes.

PSMA PET/CT imaging has emerged as a powerful tool for prostate cancer staging and recurrence assessment. Figure 2 demonstrates a prostate cancer lesion on PSMA PET/CT imaging. The addition of AI to PSMA PET/CT interpretation has accelerated progress in this area. Deep learning models trained on PSMA PET/CT datasets have achieved high performance in detecting primary tumors and metastatic lesions [24]. For example, Zhao et al. used a U-Net model to detect metastatic bone lesions and pelvic lymph node lesions with sensitivity up to 99% and 90%, respectively, which are high and comparable to physician performance [25,26] (Table 1). In addition to detection, AI-driven systems have been developed to automate tumor volume quantification and assist in staging, which can be pivotal in planning systemic therapies and radioligand treatments [24]. Efforts are also underway to integrate AI with theranostic workflows, with models designed to map PSMA expression patterns and personalize radioligand therapy planning based on imaging and clinical data [30].

In ultrasound imaging, particularly transrectal ultrasound (TRUS) and the more advanced micro-ultrasound (29 MHz), AI has begun to close performance gaps traditionally seen when compared to MRI. AI-guided systems can identify suspicious regions during TRUS exams, improving the accuracy of targeted biopsies and reducing operator dependency [33]. For example, Sun et al. developed new approaches involving 2-D CNN P-Net and 3-D CNN P-Net models for TRUS video detection with diagnostic performance of 0.85–0.89 that was superior to the TRUS 5-point Likert score system and similar in performance to the PI-RADS score system used by radiologists [26] (Table 1). The 2D P-Net was able to analyze each frame of the input image and elicit a binary decision on the presence of csPCa while the 3D P-Net was able to analyze and learn both spatial and temporal features in the TRUS video [33]. Deep learning algorithms applied to ultrasound imaging have made significant gains in prostate cancer detection, including fast and accurate segmentation of the prostate on TRUS imaging [34]. Moreover, integration of AI into ultrasound systems has facilitated streamlined workflows, faster image acquisition, and improved standardization in reporting. This has led to reduced dependence on MRI fusion techniques in some clinical scenarios, potentially increasing accessibility to advanced imaging diagnostics.

Together, these AI applications across mpMRI, PSMA PET/CT, and ultrasound underscore a major paradigm shift in prostate cancer imaging. They offer enhanced diagnostic precision, reduced variability, and improved operational efficiency—laying the foundation for more personalized and data-driven care in prostate oncology. An overview of AI technologies and applications with various imaging modalities in prostate cancer imaging is displayed in Table 2.

## 4. AI in Prostate Cancer Detection and Classification

Artificial intelligence has advanced the detection and classification of prostate cancer—especially through AI-assisted PI-RADS scoring and lesion identification. FDA-approved clinical tools, such as the Quantib Prostate and PROView, use AI and mpMRI for PI-RADS prostate density classification [10]. In a large multicase observer study by Twilt et al. involving 360 MRI examinations, readers using AI models developed with deep learning during the PI-CAI challenge had improved performance with the area under the receiver operating characteristic curve (AUROC) increasing from 0.882 to 0.916; professionals reading while using the AI model also enhanced sensitivity to 96.8% and specificity to 50.1%, compared to unassisted readings at a PI-RADS threshold of 3 or more [35] (Table 3). These findings supported the hypothesis, further showing that AI enhanced prostate cancer detection. Deep learning architectures used in the AI model are advantageous to prostate cancer imaging because they capture the fine details and features within the imaging of heterogeneous prostatic tissue. Complementing these enhancements, fully automated AI algorithms have demonstrated robust performance in lesion detection and classification across multiple centers, showing high cancer detection rates and PI-RADS distributions [36].

Beyond detection, AI-driven lesion classification and risk prediction models have been developed to outperform traditional assessments. A deep radiomics model developed by Nketiah et al. trained on multicenter MRI data achieved an AUROC of 0.91 for detecting clinically significant prostate cancer at the patient level—matching clinical PI-RADS performance [22] (Table 3), supporting its hypothesis. In this unique approach, combining radiomics with deep learning models offers a powerful advantage in detection of prostate cancer by integrating the quantitative imaging features with advanced pattern recognition, thereby enhancing the diagnostic accuracy and offering more personalized patient assessment. Explainable AI systems have further enriched interpretability, delivering visual and textual explanations that improve reader confidence and reduce interpretation time [37].

The diagnostic capability is further enhanced when integrating clinical variables with imaging data. In another study, Shu et al. developed MRI and radiomics machine learning models and found that the random forest machine learning approach had superior overall performance with AUC of 0.87 and predicted prostate cancer in the high risk group with AUC of 0.89 [38] (Table 3), comparable to clinical performance. This multimodal approach offers a broader, more accurate perspective for prostate cancer classification and supports more informed clinical decision-making with the combination of personalized assessment by incorporating radiomics data with MRI. Furthermore, the random forest machine learning approach is well-suited for prostate cancer imaging because of its ability to manage high-dimensional and heterogeneous multiparametric MRI features via the construction of an ensemble of decision trees, reducing the risk of overfitting the data and improving the predictive accuracy even in cases with small datasets. Random forests can rank the importance of radiomics and MRI features, offering insight in which biomarkers contribute most to cancer detection and characterization.

**Table 3:** Summary of AI and Prostate Cancer Imaging Studies.

Study	Imaging Modalities and Study Purpose	AI Technology	Results
Nketiah et al., 2024 [22]	MRI, Prostate Cancer Detection and Classification	Deep radiomics model	achieved an AUROC of 0.91 for detecting clinically significant prostate cancer
Twilt et al., 2025 [35]	MRI, Prostate Cancer Detection and Classification	Deep learning	improved performance with AUROC increasing from 0.882 to 0.916; professionals reading while using the AI model enhanced sensitivity to 96.8% and specificity to 50.1%, compared to unassisted readings at PI-RADS threshold of 3
Shu et al., 2023 [38]	MRI, Prostate Cancer Detection and Classification	Radiomics, random forest machine learning	developed the random forest machine learning approach with superior overall performance with AUC of 0.87 and predicted prostate cancer in the high risk group with AUC of 0.89
Litjens et al., 2014 [39]	MRI, Lesion Segmentation	CNN model	achieved median Dice scores of 0.93 for the prostate and 0.88 for the TZ of prostate MRI segmentation
Adleman et al., 2025 [40]	MRI, Tumor Volume Assessment	U-Net model	found gross tumor volume was associated with biochemical failure (hazard ratio of 1.28) and metastasis (hazard ratio of 1.34)
Bhardwaj et al., 2021 [41]	TRUS-MR fusion, biopsy target and guidance	End-to-end deep learning network	achieved a rendering rate of 14 frames per second making it compatible for live prostatic biopsy procedures
Azizi et al., 2018 [42]	Ultrasound and TeUS, biopsy target and guidance	Deep learning	achieved accuracy, sensitivity, specificity, and area under the curves results of 0.92, 0.77, 0.94, and 0.94 respectively
Kandalan et al., 2020 [43]	Dose prediction models for patients treated with VMAT, treatment planning and monitoring	Deep learning dose prediction models, Transfer learning	improved the mean Dice similarity coefficient to 0.88–0.95 and 0.92–0.96 for internal and external target institutional planning styles
Zhong et al., 2020 [44]	mpMRI, treatment planning and monitoring	AdaBoost machine learning model, radiomics	classified those with recurrence with the highest classification accuracy of 77.8% and AUC of ROC of 0.99 and 0.73 for the training and testing datasets respectively

## 5. AI in Lesion Segmentation and Volume Assessment

Accurate segmentation of the prostate gland and intraprostatic lesions is a fundamental step in the workflow of prostate cancer diagnosis and management, as it enables precise localization, measurement, and characterization of tumors. Manual segmentation by radiologists is time-consuming and subject to interobserver variability, particularly when lesions are subtle or located in challenging regions such as the transition zone (TZ). Artificial intelligence (AI) algorithms, particularly deep learning–based convolutional neural networks (CNNs), have demonstrated remarkable capabilities in automating both whole-gland and lesion segmentation in mpMRI datasets, achieving high performance levels and saving time [39,45]. For example, a CNN model was developed by Litjens et al. for prostate MRI analysis with segmentation of both the entire gland and the TZ. Tested on imaging from 104 patients, it achieved outstanding median Dice scores of 0.93 for the prostate and 0.88 for the TZ [39,45] (Table 3), demonstrating near-expert-level delineation. In addition, the model reliably distinguished slices with and without prostate tissue, reaching an average accuracy of 0.97, highlighting its potential to accelerate and standardize prostate imaging workflows while reducing the burden of manual annotation [45].

FDA-approved AI tools in clinical practice, such as ProstatID use MRI prostate imaging and incorporate 3-D CNN trained on deep learning to automatically segment the prostate and localize suspicious lesions [10]. The use of 3D-CNNs is particularly advantageous because they can capture volumetric spatial relationships across multiple MRI slices, providing a more comprehensive understanding of tumor morphology and context compared to 2D approaches. This allows for more accurate and consistent lesion identification while reducing inter-reader variability. Deep learning further enhances the system’s performance by continuously refining its pattern recognition through exposure to diverse imaging data, ultimately improving diagnostic confidence and workflow efficiency in prostate cancer evaluation.

AI-driven segmentation tools allow for the extraction of volumetric data with high spatial accuracy, enabling precise tumor volume estimation. This quantitative assessment is clinically relevant because tumor volume has been shown to correlate with disease aggressiveness, risk stratification, and prognosis [40]. For example, in a study of 187 patients led by Adleman et al., a U-Net AI-derived gross tumor volume was associated with biochemical failure (hazard ratio of 1.28) and metastasis (hazard ratio of 1.34) [40] (Table 3). The U-Net architecture excels in this setting because its encoder–decoder structure with skip connections preserves both global context and fine-grained anatomical detail, allowing for precise tumor delineation on MRI. This capability enables the model to generate clinically meaningful segmentations, improving risk stratification and supporting more informed treatment decisions for prostate cancer patients.

Furthermore, advanced AI platforms can generate three-dimensional (3D) visualizations of the prostate gland and tumor lesions, which can be integrated into clinical workflows for improved communication between radiologists, urologists, and patients. Such 3D visualizations enhance procedural planning by providing intuitive spatial orientation of lesions in relation to surrounding anatomical structures [45].

The clinical utility of AI-based segmentation extends beyond diagnosis to interventional procedures. In targeted prostate biopsy, AI-generated lesion maps can be fused with real-time ultrasound to guide needle placement with millimeter-level accuracy, reducing sampling errors and improving detection rates of clinically significant prostate cancer [46,47,48,49]. Similarly, in focal therapy planning, precise delineation of lesion boundaries and volumetric assessment are critical for determining treatment margins, minimizing damage to healthy tissue, and ensuring oncological control. AI-assisted segmentation offers reproducible and operator-independent results, which is essential for standardizing treatment planning and outcome assessment across institutions [48,49].

As these systems continue to mature, integration into multiparametric imaging platforms and interventional suites holds the potential to revolutionize the prostate cancer care pathway, from early detection to precision therapy. Nevertheless, the widespread clinical adoption of these tools will require rigorous validation across diverse patient populations, standardization of segmentation protocols, and seamless interoperability with existing imaging and treatment planning systems.

## 6. AI for Biopsy Targeting and Guidance

The integration of artificial intelligence into prostate biopsy workflows has significantly advanced the precision and efficiency of targeted sampling. A major application lies in AI-assisted fusion of TRUS and mpMRI, which enables accurate localization of suspicious lesions identified on pre-biopsy imaging. AI algorithms enhance this process by automating registration between MRI and real-time TRUS images, compensating for patient movement and organ deformation, and guiding the biopsy needle trajectory with high spatial precision. This reduces the variability associated with manual fusion techniques and has been shown to improve detection rates for clinically significant prostate cancer while minimizing unnecessary sampling of indolent lesions [50,51].

For example, an end-to-end deep learning network was used in one study by Bhardwaj et al. for TRUS-MR fusion, over 6500 images, and guided prostatic biopsy, achieving a rendering rate of 14 frames per second making it compatible for live procedures [41] (Table 3). This therefore aids live prostatic biopsy procedures in the clinical setting by reducing the false negative rate during sampling. The deep learning network was effective because it could learn complex spatial correspondences between TRUS and MRI images, enabling accurate real-time image fusion and lesion targeting. Its end-to-end design optimized both registration and visualization simultaneously, improving precision and efficiency during live prostatic biopsy procedures.

Real-time AI-guided biopsy systems have advanced further by incorporating lesion tracking, automated contouring, and dynamic adjustment of targeting based on intra-procedural feedback. By continuously analyzing TRUS image sequences, these systems can adapt to anatomical changes during the procedure and provide updated guidance to the operator. Early studies demonstrate improved biopsy yield and reduced procedure duration with such AI-assisted systems compared to conventional cognitive targeting approaches [50] (Table 3). For example, in a large clinical study by Azizi et al. with 255 biopsy cores from prostatic ultrasound data, temporal enhanced ultrasound (TeUS) is developed using deep learning to analyze ultrasound data. It achieved accuracy, sensitivity, specificity, and area under the curves results of 0.92, 0.77, 0.94, and 0.94, respectively [42], which are comparable to professional performance and can be effective aiding in the detection of prostate cancer during ultrasound-guided prostatic biopsy. The results supported the hypothesis that system integration of deep learning with ultrasound imaging and visualization enabled near-real-time analysis of TeUS data.

In addition to targeting optimization, AI models are being integrated with clinical and imaging data to predict malignancy likelihood before needle placement, enabling a more personalized approach to biopsy planning. This predictive capability can be leveraged for patient triage, optimization of the number of biopsy cores, and guidance in focal therapy planning. The combination of AI-assisted fusion imaging and predictive analytics represents a move toward precision diagnostics in prostate cancer, with the dual objectives of improving clinically significant cancer detection and reducing overdiagnosis [51,52].

## 7. AI in Treatment Planning and Monitoring

Artificial intelligence is playing an increasingly pivotal role in the personalization and optimization of prostate cancer treatment, particularly in the domains of radiation therapy planning, treatment response assessment, and prognostication. In radiation oncology, AI-based contouring algorithms have demonstrated the ability to automatically delineate target volumes and organs at risk on multiparametric MRI and CT scans with high concordance to expert annotations. This automation not only reduces inter-observer variability but also significantly shortens planning time, allowing for more efficient workflow integration. Deep learning models can further assist in dose optimization by predicting optimal radiation dose distributions that balance tumor control with organ preservation, thereby improving both oncologic outcomes and quality of life for patients [53,54].

Transfer learning has become a transformative approach in radiotherapy, allowing AI models to leverage knowledge from existing datasets and efficiently adapt to new clinical contexts with limited data. In prostate cancer radiotherapy planning, this method has shown remarkable potential to bridge institutional and protocol-specific variations. In a seminal study, Kandalan et al. demonstrated that transfer learning could effectively recalibrate deep learning dose prediction models for patients treated with VMAT, achieving high accuracy even when trained on as few as 16 target cases [43,54]. By transferring learned representations from one planning style to another, the models substantially reduced prediction errors and improved dose consistency; it improved the mean Dice similarity coefficient to 0.88–0.95 and 0.92–0.96 for internal and external target institutional planning styles [54] (Table 3). This innovation highlights how transfer learning can accelerate the clinical deployment of AI-driven planning tools, enhancing precision and personalization in prostate cancer radiotherapy.

Beyond treatment initiation, AI tools are increasingly utilized to assess therapy response and disease progression in patients on active surveillance or undergoing focal and systemic therapies. By analyzing serial imaging data in conjunction with clinical and biomarker information, AI algorithms can detect subtle morphologic or functional changes that may indicate early progression, enabling timely intervention. For example, AI-driven radiomics approaches applied to multiparametric MRI have shown promise in differentiating responders from non-responders to radiation therapy in the context of androgen deprivation therapy or focal ablation techniques [54]. With its ability to enhance detection by iteratively improving focus on challenging-to-classify lesions and discovering subtle patterns, the adaptive Boosting (AdaBoost) machine learning model was developed by Zhong et al. to classify those with recurrence with the highest classification accuracy of 77.8% and AUC of ROC of 0.99 and 0.73 for the training and testing datasets, respectively, [44] (Table 3). These results showed potential for the MRI-based radiomics machine learning model in the future of precision medicine.

AI is also being leveraged for prognostication by integrating imaging features, histopathology, genomics, and clinical parameters into predictive models that estimate individualized risks of recurrence, metastasis, and cancer-specific mortality. These models can provide clinicians with actionable insights to guide decisions on treatment intensification, de-escalation, or transition to palliative strategies. By offering objective, data-driven predictions, AI has the potential to complement existing nomograms and risk stratification systems, ultimately enabling more precise and patient-centered care [55,56].

## 8. Challenges, Disadvantages, and Limitations

Despite the rapid progress and promising clinical applications of AI in prostate cancer imaging and management, several challenges and limitations must be addressed before widespread adoption can be realized. One of the primary disadvantages is the limited availability of large, high-quality, and well-annotated datasets for training robust algorithms. Even when such datasets exist, their heterogeneity in terms of imaging protocols, scanner types, patient demographics, and annotation standards can hinder model performance and reproducibility across institutions. This variability underscores the need for harmonization of imaging acquisition and reporting standards to facilitate multicenter collaborations and improve data interoperability [57,58].

Another significant potential disadvantage is the lack of rigorous external validation, which limits the generalizability of AI models. Many studies report high performance on internal datasets but fail to replicate these results when tested on external cohorts, raising the risk of overfitting and inflated accuracy metrics. Without consistent external validation and benchmarking across diverse populations, the clinical reliability of AI tools remains uncertain [59].

Ethical considerations also play a central role, particularly regarding algorithmic bias. Models trained on datasets that underrepresent certain racial, ethnic, or socioeconomic groups may inadvertently perpetuate healthcare disparities by performing suboptimally in these populations. This risk calls for deliberate efforts to ensure diversity and representativeness in training datasets, as well as transparency in model design and decision-making processes [60].

Regulatory and medico-legal frameworks for AI in healthcare are still evolving. Unlike traditional medical devices, AI systems—especially those employing continuous learning—pose unique challenges in terms of regulatory approval, post-market surveillance, and liability in the event of diagnostic errors. Guidelines from regulatory agencies and professional societies will be essential to ensure safe and accountable clinical deployment [61].

Despite the promise of artificial intelligence (AI) and deep learning for enhancing prostate cancer imaging, one of the most significant challenges lies in the “black box” nature of these algorithms. Deep learning models, particularly convolutional neural networks, often provide highly accurate outputs without transparent reasoning or easily interpretable decision pathways. This opacity poses a barrier to clinical trust and adoption, especially in domains such as prostate cancer imaging where nuanced decisions affect patient management and outcomes.

A central concern is the potential for bias at multiple stages of the AI workflow. At the pre-processing stage, training datasets may inadvertently underrepresent certain patient demographics, disease subtypes, or imaging acquisition protocols, leading to systematic biases in algorithm performance. For example, models trained predominantly on data from one vendor or institution may not generalize well to other clinical settings, introducing variability in lesion detection or classification accuracy.

During post-processing tasks, such as lesion segmentation, algorithmic bias can manifest as either over- or under-segmentation of suspicious regions. This may alter lesion size estimation, affect PI-RADS scoring, and ultimately impact biopsy or treatment decisions. Furthermore, segmentation errors are not always apparent to the interpreting radiologist, creating a hidden layer of vulnerability that can propagate downstream into clinical care.

The interpretation stage also carries risks. Because deep learning models lack inherent explainability, subtle confounding factors—such as scanner noise, motion artifacts, or incidental findings—may influence predictions in ways that are opaque to the user. Radiologists are then left with the challenge of reconciling the algorithm’s output with their own clinical judgment, without clear insight into why the AI model arrived at a given conclusion. This lack of interpretability not only undermines confidence but may also perpetuate biases if unrecognized patterns in the data disproportionately affect certain patient groups.

Addressing these drawbacks requires ongoing efforts toward explainable AI, standardized validation frameworks across diverse populations, and robust human–AI collaboration strategies. Without transparency, the risk remains that deep learning could reinforce inequities and introduce new errors in prostate cancer imaging, counteracting the very advances it aims to deliver.

Finally, the integration of AI into routine clinical workflows faces practical obstacles, including interoperability with existing health information systems and user interface design. Even the most accurate algorithms will fail to make an impact if they are not perceived as trustworthy, intuitive, and complementary to clinical expertise. Effective implementation will require multidisciplinary collaboration between AI developers, clinicians, and health system administrators to align technological capabilities with real-world clinical needs [62], especially in underserved regions.

## 9. Future Directions and Opportunities

The future of artificial intelligence in prostate imaging centers on the integration of multimodal data sources, combining imaging with genomic, proteomic, and clinical information to advance personalized medicine. By fusing multiparametric MRI, PET, and ultrasound data with molecular biomarkers, AI systems are expected to enhance risk stratification, refine diagnostic accuracy, and tailor treatment recommendations to individual patient profiles [63,64]. This integrative approach may improve prognostic modeling and provide clinicians with comprehensive decision-support tools that incorporate diverse patient-specific factors. A summary of future directions and impact of AI applications in prostate cancer imaging are displayed in Table 4.

Federated learning offers a promising solution to data privacy challenges by enabling AI models to be trained collaboratively across multiple institutions without requiring the sharing of raw patient data. This decentralized learning approach can enhance model robustness and generalizability by leveraging diverse datasets, while maintaining patient confidentiality [65]. Successful deployment of federated learning will depend on coordinated efforts among academic centers, industry partners, and regulatory bodies.

AI-driven decision-support platforms hold potential to transform multidisciplinary care by synthesizing complex data streams into actionable clinical insights. These systems could streamline diagnostic workflows, optimize treatment planning, and predict outcomes, facilitating precision oncology tailored to each patient’s unique disease biology and comorbidities.

In addition to image analysis, AI systems employing natural language processing (NLP) are being used to extract structured data from unstructured radiology reports. This facilitates the creation of large, annotated datasets necessary for training AI models and supports real-time clinical decision support systems [17]. For instance, NLP tools have been successfully applied to classify radiology reports based on PI-RADS scores and biopsy recommendations, allowing for rapid cohort assembly and analysis [18]. The integration of NLP with imaging AI tools holds promise for improving communication, workflow efficiency, and reporting standardization.

AI and digital twin technology are increasingly being applied to prostate cancer imaging to improve early detection, diagnosis, and risk stratification. By creating a patient-specific digital replica of the prostate, digital twins can integrate multiparametric MRI, ultrasound, and histopathology data with AI-driven models to simulate disease behavior and guide clinical decision-making. These systems can help address inter-observer variability in imaging interpretation, particularly in lesion characterization and Gleason grading, by providing standardized, reproducible outputs that align closely with expert assessment [66,67,68]. Moreover, AI-enabled digital twins allow continuous refinement through feedback loops, enabling them to predict tumor progression and treatment response with high precision. Such advancements highlight the potential of AI-driven digital twins to reduce diagnostic uncertainty, enhance individualized care, and ultimately improve clinical outcomes in prostate cancer detection.

Artificial intelligence in medical imaging has progressed toward powerful large models and vision–language models (VLMs) capable of generalizing across modalities and tasks. VLMs represent a transformative advance, enabling systems that can integrate visual perception with natural language understanding [67,68,69,70]. In medical imaging, these models move beyond siloed single-modality applications, supporting tasks such as report generation, visual question answering, and image–text grounding, while also demonstrating adaptability to new tasks through zero- and few-shot learning. Recent reviews describe the trajectory from early fusion-based architectures to large multimodal foundation models with application of VLM-based medical image analysis [71], emphasizing strategies such as contrastive pretraining, generative alignment, and prompt-based tuning. Emerging work in 3D medical VLMs further illustrates their ability to process volumetric scans alongside textual reports, creating opportunities for richer, context-aware prostate imaging analysis.

Looking forward, VLMs are expected to play a pivotal role in advancing precision oncology. Vision–language foundation models, when combined with multimodal data sources such as genomics, pathology, and clinical notes, can provide more holistic insights into prostate cancer detection, characterization, and treatment planning. These systems may evolve into interactive diagnostic assistants capable of answering case-specific questions such as the presence of extra-prostatic extension, guiding biopsy targeting, or drafting structured reports.

Crucial to the responsible adoption of AI is comprehensive education and training for clinicians. A thorough understanding of AI methodologies, strengths, and limitations will empower clinicians to critically interpret algorithm outputs and integrate them effectively into patient care [62]. Collaborative development between clinicians and data scientists is essential to ensure that AI tools are clinically relevant and seamlessly integrated into existing workflows in the future.

Continued multidisciplinary collaboration, rigorous validation studies, and alignment with evolving regulatory frameworks will be necessary to realize AI’s full potential to aid in prostate cancer care, ultimately improving patient outcomes and advancing the standard of care [66].

## 10. Conclusions

Artificial intelligence has rapidly emerged as a transformative force in prostate imaging, significantly enhancing the accuracy, efficiency, and reproducibility of cancer detection, lesion characterization, biopsy guidance, and treatment planning. Advanced AI technologies—including machine learning, deep learning, and radiomics—are now being applied across a spectrum of tasks, from lesion detection and risk stratification to automated prostate and tumor segmentation, targeted biopsy guidance, and radiation therapy optimization. Various developed AI models including 2-D and 3-D CNNS, U-Net, V-Net, P-Net, random forest, and adaptive Boosting models, have aided in prostate cancer detection, classification, segmentation, volume delineation, prognosis, and biopsy guidance.

These advancements hold substantial potential to improve diagnostic confidence, reduce interobserver variability, and personalize therapeutic strategies, thereby advancing prostate cancer management toward precision medicine [33,67].

AI applications extend across multiple imaging modalities, including multiparametric MRI, PSMA PET/CT, and ultrasound, each benefiting from automated analysis pipelines that enhance clinical workflows and decision-making. The integration of AI into PSMA PET and PET/CT imaging has improved lesion detection and metastasis assessment, while AI-enhanced ultrasound systems have expanded access to high-quality diagnostic capabilities in settings with limited subspecialty expertise.

Looking forward, the integration of AI-augmented tools into clinical workflows promises to revolutionize prostate cancer care by enabling comprehensive data synthesis that combines imaging, genomics, and clinical information. Such innovations can facilitate more informed decision-making, optimize treatment efficacy, and improve patient outcomes. However, realizing this vision requires overcoming existing challenges related to data heterogeneity, algorithm generalizability, ethical considerations, and regulatory oversight [53].

Looking ahead, the next generation of AI in prostate imaging will likely be characterized by multimodal models that integrate imaging, genomics, pathology, and clinical data to deliver personalized risk prediction, treatment selection, and outcome forecasting. Federated learning and collaborative, privacy-preserving model development could accelerate innovation while safeguarding patient data. Ultimately, realizing the full potential of AI-augmented prostate cancer care will require sustained multidisciplinary collaboration among clinicians and data scientists, supported by high-quality prospective evidence. With thoughtful implementation, AI has the capacity to extend specialist-level diagnostic expertise to underserved regions, advance precision medicine, and improve outcomes for patients worldwide. Through such concerted efforts, AI can assist clinicians in prostate cancer care, delivering precision diagnostics and personalized therapies that ultimately improve survival and quality of life for patients [59,62].

## Figures and Tables

**Table 2:** Summary of AI Technologies and Applications in Prostate Cancer Imaging.

Domain	Imaging Modalities	AI Technologies	Applications
Detection & Classification	mpMRI, PSMA PET/CT, PET/MRI, TRUS	Machine learning, deep learning (CNNs), radiomics	AI-assisted PI-RADS scoring, lesion detection, risk prediction
Lesion Segmentation & Volume Assessment	mpMRI, PET, Ultrasound	Deep learning (U-Net, CNN-based segmentation), radiomics	Automated gland and lesion segmentation, tumor volume estimation, 3D visualization
Biopsy Targeting & Guidance	TRUS, MRI-TRUS fusion	Deep learning, reinforcement learning, real-time AI-assisted navigation	AI-assisted fusion, real-time biopsy guidance, target selection
Treatment Planning & Monitoring	mpMRI, PSMA PET, Ultrasound	Deep learning, ML-based outcome prediction models	Radiation therapy contouring and dose optimization, treatment response monitoring, prognostication

**Table 4:** Summary of Future Directions and Impact of AI Applications in Prostate Cancer Imaging.

Focus Area	Key Advancements	Expected Impact
Multimodal Data Integration	Combines imaging (mpMRI, PET, ultrasound) with genomic, proteomic, and clinical data for personalized medicine.	Enhances diagnostic accuracy, risk stratification, and individualized treatment recommendations.
Federated Learning	Decentralized AI training across institutions without sharing raw data.	Improves model robustness and generalizability while maintaining patient privacy; collaboration across academia, industry, and regulators.
AI-Driven Decision Support Systems	Integrates multimodal data into actionable insights for clinicians.	Streamlines diagnostics, optimizes treatment planning, and predicts outcomes for precision oncology.
Natural Language Processing (NLP)	Extracts structured data from unstructured radiology reports (e.g., PI-RADS classification, biopsy recommendations).	Enables rapid dataset creation, standardizes reporting, improves workflow efficiency, and enhances communication.
Digital Twin Technology	Creates a patient-specific virtual model integrating MRI, ultrasound, histopathology, and AI models.	Reduces inter-observer variability, standardizes Gleason grading, predicts progression and treatment response, and personalizes care.
Large AI and Vision–Language Models (VLMs)	Foundation models integrating imaging and text; support report generation, question answering, and image–text alignment.	Enables generalization across modalities, and advances context-aware prostate imaging analysis.
Precision Oncology with VLMs	Combines VLMs with genomics, pathology, and clinical notes for holistic prostate cancer analysis.	Enables interactive diagnostic assistants that can guide biopsy targeting, detect extra-prostatic extension, and draft structured reports.

## Data Availability

No new data were created or analyzed in this study. Data sharing is not applicable to this article.
